# Supplementary material for: MetaQTL: a package of new computational methods for the meta-analysis of QTL mapping experiments
Source: BMC Bioinformatics. 2007 Feb 8;8:49. doi: 10.1186/1471-2105-8-49 (PMC1808479; doi:10.1186/1471-2105-8-49)
Supplement: Additional File 7 — MetaQTL Package : jar file and tutorial. This Zip archive contains both the MetaQTL JAR file and the files of the tutorial. [file 1471-2105-8-49-S7.zip › org.inra.metaqtl/doc/org/metaqtl/Qtl.QTLStats.html]

Qtl.QTLStats


|  |  |  |
| --- | --- | --- |
| **Overview** \| **Package** \| **Class** \| **Use** \| **Tree** \| **Deprecated** \| **Index** \| **Help** | |  |
| **PREV CLASS**   **NEXT CLASS** | **FRAMES**    **NO FRAMES**     **All Classes** |  |
| SUMMARY: NESTED \| FIELD \| CONSTR \| METHOD | DETAIL: FIELD \| CONSTR \| METHOD |  |


---


## org.metaqtl Class Qtl.QTLStats

```
java.lang.Object
  org.metaqtl.Qtl.QTLStats
```

**All Implemented Interfaces:**: java.io.Serializable

**Enclosing class:**: Qtl

---

``` public class Qtl.QTLStats extends java.lang.Object implements java.io.Serializable ```

This class stores the usual statistics for the QTL.

**See Also:**: Serialized Form

---

| **Field Summary** | |
| --- | --- |
| `double` | `additive`             The additive effect of the QTL. |
| `double` | `dominance`             The dominance effect of the QTL. |
| `double` | `lod`             The lod score of the QTL. |
| `double` | `rsquare`             The r-square, i.e the proportion of variance explained by the QTL. |


**Constructor Summary**

`Qtl.QTLStats()`


| **Method Summary** | |
| --- | --- |
| `Qtl.QTLStats` | `getCopy()` |

**Methods inherited from class java.lang.Object**

`clone, equals, finalize, getClass, hashCode, notify, notifyAll, toString, wait, wait, wait`

**Field Detail**

### rsquare

```
public double rsquare
```

:   The r-square, i.e the proportion of variance
    explained by the QTL.

---


### additive

```
public double additive
```

:   The additive effect of the QTL.

---


### dominance

```
public double dominance
```

:   The dominance effect of the QTL.

---


### lod

```
public double lod
```

:   The lod score of the QTL.


**Constructor Detail**

### Qtl.QTLStats

```
public Qtl.QTLStats()
```


**Method Detail**

### getCopy

```
public Qtl.QTLStats getCopy()
```

:   **Returns:**


---


|  |  |  |
| --- | --- | --- |
| **Overview** \| **Package** \| **Class** \| **Use** \| **Tree** \| **Deprecated** \| **Index** \| **Help** | |  |
| **PREV CLASS**   **NEXT CLASS** | **FRAMES**    **NO FRAMES**     **All Classes** |  |
| SUMMARY: NESTED \| FIELD \| CONSTR \| METHOD | DETAIL: FIELD \| CONSTR \| METHOD |  |


---
